# Supplementary material for: Cost-effectiveness requirements for implementing artificial intelligence technology in the Women’s UK Breast Cancer Screening service
Source: Nat Commun. 2023 Sep 30;14:6110. doi: 10.1038/s41467-023-41754-0 (PMC10542368; doi:10.1038/s41467-023-41754-0)
Supplement: Supplementary file 2 — Description of Additional Supplementary Files [file 41467_2023_41754_MOESM2_ESM.pdf]

Description of Additional Supplementary Files

File name: Supplementary Data 1

Description: Maximum Reimbursable Price of the Mia Technology estimation

File name: Supplementary Data 2

Description: Setup cost analysis

File name: Supplementary Data 3

Description: Maintenance cost analysis @£17,000 per year

File name: Supplementary Data 4

Description: Locum analysis

File name: Supplementary Data 5

Description: Brest cancer incidence from age 19

File name: Supplementary Data 6

Description: Probability of NPI membership by tumour size

File name: Supplementary Data 7

Description: Women's life table 2016-18(Suplementary Data 5).
